# Patient-Reported Disease Presentation, Interventions, and Outcomes in United States Pregnancies Affected by Alloimmunization at Risk of Hemolytic Disease of the Fetus and Newborn

**DOI:** 10.3390/children12070822

**Published:** 2025-06-22

**Authors:** Molly R. Sherwood, Bethany M. Weathersby, Marion E. Granger Howard, Kara B. Markham

**Affiliations:** 1 Allo Hope Foundation, 1655 N McFarland Blvd, Suite 256, Tuscaloosa, AL 35406, USA; 2 Department of Epidemiology, University of South Carolina, Columbia, SC 29208, USA; 3 Department of Obstetrics and Gynecology, University of Cincinnati, Cincinnati, OH 45267-0526, USA

**Keywords:** alloimmunization, fetal hydrops, hemolytic anemia, hemolytic disease, intrauterine transfusion, Rh disease

## Abstract

Background/Objectives: Pregnancies complicated by red cell alloimmunization can progress to hemolytic disease of the fetus and newborn (HDFN), requiring close monitoring and timely intervention to prevent fetal/neonatal morbidity and mortality. This study investigated disease presentation, interventions, and outcomes in respondents with a history of alloimmunized pregnancy. Methods: This was a retrospective cross-sectional survey study administered online to alloimmunized patients between November 2022–February 2023. A total of 127 participants reported on 200 alloimmunized pregnancies. Distribution of pregnancy characteristics, antibodies and titers, monitoring, treatments and fetal outcomes were described and stratified where appropriate by fetal antigen status and disease severity. Outcomes and management practices in subsequent pregnancies following fetal loss to HDFN are reported. Results: Multiple antibodies were present in 42% of pregnancies with known antibody type (80/192). Titers reached critical levels (any titer for Anti-K; ≥16 for all other antibodies) in 71% (125/176) of pregnancies where titer was reported. Among fetal antigen positive pregnancies with critical titers, intrauterine transfusions were conducted in 40% (42/106), intravenous immunoglobulin was administered in 14% (15/106), plasmapheresis in 7% (7/106), and phenobarbital in 9% (9/106). Complications from transfusion were reported in 38% of pregnancies receiving intrauterine transfusion (17/45). Fetal death due to HDFN or complications from intrauterine transfusion was reported in 9% of antigen positive pregnancies with critical titers (10/106) and 16% of pregnancies receiving intrauterine transfusion (7/45). Conclusions: Disease presentation and severity complements previous research in this disease population, however, monitoring practices were diverse. Fetal death and intrauterine complication rates were higher than those previously reported in large international referral centers. Development of best practices and centralized referral centers may improve disease outcomes.

## 1. Introduction

Maternal red cell alloimmunization is an obstetric condition whereby a person of childbearing potential develops red cell antibodies after exposure to foreign blood, usually during pregnancy or transfusion. These antibodies can cross the placenta and destroy fetal red blood cells with the corresponding antigen, resulting in hemolytic disease of the fetus and newborn (HDFN) in the child. HDFN causes fetal anemia and neonatal anemia and hyperbilirubinemia. If not treated promptly through intrauterine blood transfusions (IUTs) and/or aggressive phototherapy and blood transfusion in the newborn period, HDFN can progress to hydrops, bilirubin induced encephalopathy, heart failure and death [1,2].

The introduction of Rh immune globulin in 1968 contributed to a significant reduction in what was then termed “Rh disease”. Maternal red cell alloimmunization is now considered a rare disease in developed countries, with clinically significant antibodies occurring in 0.7–1.7% of pregnancies in the United States [3,4,5]. This condition still occurs in instances of missed Rh immune globulin administration [6], in occasional cases where Rh immune globulin is ineffective or the dosing is insufficient [7], and in the case of the more than 50 other red cell antigens which are known to cause HDFN but for which no prophylaxis exists.

Because many maternal fetal medicine practitioners will manage a handful of cases of alloimmunization in the lifetime of their practice, an opportunity to develop skill in disease management is lacking [8]. IUTs are currently the only in-utero treatment for anemic fetuses suffering from HDFN. Given its rarity in the (U.S.) and the broad geographic distribution of alloimmunized patients, many practitioners are not afforded the opportunity to develop required expertise in the conduct of IUTs. Other treatments to delay the onset of fetal anemia, such as plasmapheresis and intravenous immunoglobulin (IVIG) [9,10], as well as maternal treatments such as phenobarbital to reduce the need for newborn exchange transfusion have not been widely studied and consistently implemented in practice [11].

Research in the alloimmunized population is also limited by its rarity. Most database studies conducted to-date are limited in evaluable metrics and report on prevalence or mortality [4,5,12]. Others face natural limitations of sample size [13,14], or are studies from large referral centers representing highly skilled care which may not reflect the entire U.S. care profile [15,16]. The hospital systems that research alloimmunization and HDFN most prolifically are in countries that serve as the central referral site, which does not properly represent the U.S. healthcare system [17,18,19]. The objective of this report was to summarize disease presentation, management, outcomes, and severity in alloimmunized pregnancies at risk for HDFN in the U.S., the knowledge of which may prove useful in reducing morbidity and mortality from this disease.

## 2. Materials and Methods

This study was a retrospective cross-sectional structured survey study approved by WIRB-Copernicus Group (IRB tracking number 20224681). Allo Hope Foundation staff invited alloimmunized women to participate in the voluntary survey through a closed online patient support group, social media, and electronic newsletters. Recruitment took place between November 2022 and February 2023. Participants were compensated with a gift card. Inclusion criteria for this nationwide survey included adults living in the U.S. with a previous alloimmunized pregnancy lasting more than 12 weeks that had completed (resulting in live birth, fetal death or neonatal death). Participants must have had at least one red cell antibody known to cause HDFN [20], even if their fetus was determined to be antigen negative. Participants were not eligible to participate if they were currently pregnant with their first alloimmunized pregnancy.

The survey was developed by a multidisciplinary team of clinicians, patients and researchers. The survey collected data related to disease severity, fetal and neonatal management and outcomes, mental health impact and effect of red cell alloimmunization/HDFN on daily life. The authors utilized The Checklist for Reporting Results of Internet E-Surveys in preparation and design of this survey [21]. Adaptive questioning was employed to eliminate irrelevant questions for certain respondents (e.g., a participant who reported fetal death was not asked questions about neonatal care). The questionnaire was administered and data collected and stored through Qualtrics, in a closed survey where each interested participant was provided with a unique one-time use link to prevent multiple entries or bot activity. No protected health information was gathered or stored. In order to issue payment via e-mail without sacrificing anonymity, participants were directed to a secondary survey after completion where they provided their e-mail address.

The survey gathered informed consent by asking each participant to read the informed consent form and agree that they consent and are willing to proceed with the survey, and participants were told the length and purpose of the investigation. The complete questionnaire is available upon request. In addition to providing basic demographics and delivery details, participants answered questions for each alloimmunized pregnancy including antibody type and titers, fetal antigen status and method of determining fetal antigen status, and timing of diagnosis. Frequency of titer assessments and Middle Cerebral Artery (MCA) Doppler scans, when applicable, was reported, as well as antenatal interventions and any resultant complications. For participants who received IUTs, the highest MCA Doppler Multiples of the Median (MoM) value, number of IUTs, and gestation at first and last IUT were recorded. Consequences of HDFN in the fetus were reported as well as neonatal treatment and outcomes, quality of care, quality of life impact and psychosocial burden.

Categorization of variables was designed to reflect current clinical thresholds and guidelines for HDFN. Severe disease was defined as an antigen positive pregnancy receiving IUT prior to 24 weeks, plasmapheresis, IVIG, or the presence of fetal hydrops or death. Investigators categorized a pregnancy as having critical titers if the respondent reported a highest known titer of 16 or above for all antibodies except Kell (all Kell pregnancies were considered to have a critical titer). In the instances where the respondent did not report their highest known titer, the investigators deferred to the respondent’s self-report of critical versus non-critical titer.

Only completed questionnaires were included for analysis. Most questions allowed a response of “don’t know” if the respondent failed to recall the answer to the question to reduce risk of recall bias. The distribution of pregnancy specific characteristics, including antibody variants, the time at which titers reached critical level, prenatal treatments, fetal outcomes, and mode of delivery were described for all pregnancies, as well as by antigen status if known and among those with critical or non-critical titers or those requiring IUTs when appropriate. Categories were grouped to minimalize identifiability. Descriptive data analysis was conducted in SAS 9.4.

## 3. Results

A total of 172 potentially eligible respondents requested a unique survey link to complete the survey and 130 initiated the survey (75.6%). Of the 130 surveys initiated, 127 surveys completed all required sections (97.7%). These 127 alloimmunized patients reported on 200 alloimmunized pregnancies (Table 1). “Don’t know” responses varied by question and were excluded from analysis (e.g., RBC antibody type was known in 192/200 pregnancies (96.0%); fetal antigen status was known in 167/200 pregnancies (83.5%). Patients reported on alloimmunized pregnancies between 2003–2022 with the most in 2020 (*n* = 47) followed by 2021 (*n* = 40). The majority of patients were non-Hispanic white (91.3%), had bachelor’s degrees or higher (61.4%), reported family income at or above $52,200 (81.9%), and carried private insurance (64.6%). Most participants had two or three living children (42.9% and 29.4%) and reported one or two previous alloimmunized pregnancies (61.4% and 26.0%).

Maternal antibody identification was known in 192 of 200 alloimmunized pregnancies (96.0%); antibody titers were known in 176 of 200 alloimmunized pregnancies (88.0%) (Table 2). Multiple antibodies were reported in 41.7% of pregnancies (80/192). Of pregnancies with only one antibody, Anti-K was most prevalent (*n* = 38; 19.8% of full sample), followed by Anti-E (*n* = 33; 17.2%) and Anti-D (*n* = 27; 14.1%). Fetal antigen status was known in 167 of 200 alloimmunized pregnancies (83.5%). Among these, antigen status was determined by paternal antigen typing in 121/167 (72.5%), neonatal direct agglutination test in 77/167 (46.1%), cell free fetal DNA in 27/167 (16.2%), amniocentesis in 14/167 (8.4%), symptoms alone in 10/167 (6.0%), and other methods in 2/167 (1.1%) and not reported or don’t know in 6/167 (3.6%) (note that often multiple modalities were used). Titers reached critical levels in 82.5% of antigen positive pregnancies (99/120) and 51.4% of antigen negative pregnancies (18/35).

Pregnancy monitoring, interventions and fetal outcomes are reported in Table 3. Once titers reached critical levels, MCA Doppler ultrasounds were most often conducted weekly (59.4%) or every two weeks (24.5%). Monitoring with nonstress tests (NSTs) and biophysical profiles (BPPs) was reported across antigen positive pregnancies with a critical titer (77.4% and 59.4%, respectively), antigen positive pregnancies with below critical titers (65.0% and 50.0%), and antigen negative pregnancies (67.5% and 40.0%). Interventions in antigen positive pregnancies with critical titers included IVIG (14.2%), plasmapheresis (6.6%), antenatal phenobarbital (8.5%), and antenatal corticosteroids (50.0%).

Intrauterine transfusions were conducted in 22.5% of the full cohort (45/200) and 39.6% of antigen positive pregnancies with critical titers (42/106). Pregnancies receiving IUTs most commonly reported two (27.9%) or three (23.3%) IUTs. Eighteen complications from IUT were reported in 17 (38.6%) of pregnancies receiving IUTs including fetal death in five cases, fetal bradycardia/distress resulting in emergency c-section in three, maternal hemorrhage or abruption in three, inability to complete procedure in two, fetal distress in two, chorioamnionitis in one, preterm labor in one, and chorion amnion separation in one.

Fetal complications in the full sample included anemia on MCA doppler ultrasound (29.0%), ascites (5.0%), hydrops (2.1%), and in utero demise (5.5%), with 10 out of 11 fetal deaths attributed to HDFN. The single fetal death unrelated to alloimmunization was related to Trisomy 18. Fetal deaths due to HDFN are further profiled in Table 4. Five of the ten fetal deaths were the respondent’s first alloimmunized pregnancy. Antibody titers among pregnancies resulting in fetal death ranged from 64 to greater than 16,384. A total of 10 subsequent antigen positive pregnancies were reported by participants who experienced a fetal death due to HDFN. Of these, one was antigen negative, one resulted in fetal death, and the remaining eight resulted in a live birth.

Thirty-one of the 175 pregnancies with known fetal antigen status presented with severe disease (18% of all pregnancies, 23% of fetal antigen positive pregnancies (31/134)). Mode and reason for delivery is described in Table 5. Among the full sample, 125 (66.1%) resulted in a vaginal delivery and 64 (33.9%) in cesarean section. Emergency cesarean was required in 21 deliveries (10.5%) and in 10 out of 38 live birth pregnancies requiring IUT (26.3%). Gestational age at birth was most commonly 37–38 weeks in antigen positive pregnancies (48.4%) and 34–36 weeks (54.1%) in pregnancies receiving IUT.

## 4. Discussion

This is a unique glimpse into disease management and outcomes in U.S. alloimmunized pregnancies spanning across treatment centers. The findings demonstrate the variability in disease trajectory and treatment outcomes on a national scale that can be used to inform managing providers of potential outcomes and treatment options in the absence of a high case volume. An opportunity presents itself to reflect on these findings both as individual cases detailing procedural complications and fetal death and comprehensively to understand broad disease presentation and treatment courses. The results of this study indicate antibody distributions similar to those described in previous literature with D, K, and E being among the most prevalent [4,5,18,22]. Previously conducted research indicates that 56–75% of patients with clinically significant red cell antibodies have antigen positive fetuses at risk of HDFN [17,18,23], and 22–23% of at-risk fetuses with critical titers receive at least one IUT [18,24]. These findings are comparable to our population where 78% of pregnancies were antigen positive and 33% of antigen positive fetuses received IUTs.

Published survival rates in fetuses requiring IUT for HDFN range from 93–97% in developed countries [17,18,25]. These are representative of large referral centers in Europe and may not be a reflection of the diverse care practices available in other countries. Indeed, the fetal survival rate for fetuses receiving IUT in this study was 84%. In countries with centralized referral centers, IUT complication rates per fetus have been reported to be approximately 3–5% [25,26], but 38% of pregnancies requiring IUT in this study incurred a complication. Previous literature has demonstrated that initial competence in performing IUTs is reached after 30–50 procedures with maintenance of 10 procedures annually [27]. This may not be attainable, however, without establishing a referral program to high-volume fetal centers.

Alloimmunized pregnancy monitoring practices can also be sharpened in response to these findings. Titers reached critical levels frequently in both affected (antigen positive) and unaffected (antigen negative) pregnancies, indicating that titers alone are not sufficient to intuit fetal antigen status. Amniocentesis was used sparingly in this population, indicating a potential shift towards noninvasive screening through cell free DNA to avoid unnecessary pregnancy risk and worsening of alloimmunization which can precipitate after invasive procedures, although since this dataset includes pregnancies from prior to the use of cffDNA for fetal antigen typing in the U.S., this is best evaluated in prospective literature [28,29]. The majority of pregnancies with critical titers received weekly MCA Doppler scans, a useful snapshot considering current guidelines do not propose definitive intervals [20]. It is notable that 44.7% (17/38 pregnancies requiring IUT which reported their highest MCA scan value) of the pregnancies receiving IUTs had an MCA Doppler MoM value at or above 1.7. This threshold is well above the recommended threshold of 1.5 MoM. Waiting to conduct IUTs until fetal anemia progresses to hydrops has been demonstrated to result in more procedure complications and a higher likelihood of fetal death [30].

While severe disease occurs in only a subset of the alloimmunized patient population, anticipating the risk of severe disease and monitoring accordingly will ensure the highest likelihood of fetal survival. Of the pregnancies which resulted in fetal death, half of these were the mother’s first alloimmunized pregnancy. Previous research has supported the risk of developing significant HDFN in a first alloimmunized Anti-D pregnancy at a rate of approximately one third [31]. Despite this, many institutions do not offer IVIG or plasmapheresis in first alloimmunized pregnancies, reserving these therapies for patients who have experienced previous fetal death due to HDFN. One might presume that the pregnancies resulting in fetal death were extremely severe and that any subsequent pregnancy would result in a similar outcome. It is notable that, of the ten pregnancies that followed a fetal loss due to HDFN, one was antigen negative, one resulted in fetal death (with maternal antibody titers above 16,384), and the remaining eight antigen positive fetuses resulted in live birth. Eight of nine of these pregnancies began MCA Doppler ultrasounds prior to 16 weeks, a practice which has demonstrated accuracy in the hands of skilled sonographers but is not common practice at many high-risk perinatal ultrasound offices [32]. Attention should be given to vigilant monitoring and thoughtful treatment planning with preventative treatments to delay onset of fetal anemia when appropriate.

While convenience sampling is common in rare disease research [33,34], future efforts may require large multi-center engagement with or without retrospective electronic medical record review or prospective registry studies to achieve adequate probability sampling. Future examinations of the impact of multiple antibodies on disease severity may be an important consideration for management practices. It is possible that patients with severe or unusual disease trajectories would be more likely to complete the survey, although investigators were thoughtful to utilize inclusive language in study recruitment materials in an attempt to reduce this risk by explicitly inviting participants with any severity of disease history including mothers with antigen negative pregnancies to participate. Demographics in this sample skew towards white, educated, high-income families with private insurance coverage which is not a generalizable sample. This may be a reflection of the fact that women were recruited for this study from a patient support group for a complex rare disease, which may select for people with advanced medical literacy. It should be considered that these outcomes may reflect a patient pool who is more likely to be comfortable advocating for care, have resources to change providers, and have more social support. One may infer that outcomes in a population more representative of the average U.S. family could be less optimal.

Like any survey, this investigation is subject to recall bias. However, it is the experience of the authors and in rare disease research that such patients become well educated and strong advocates for their care [35,36]. Such motivated patients may be likely to maintain records and to properly recall their medical care and with extensive learned expertise; indeed 98% of surveys were completed in this study. The degree of missingness in some variables may in and of itself reflect inconsistent monitoring or lack of clinician-patient collaboration. For example, missingness for fetal antigen status may be because women report that their providers did not test fetal antigen status at birth and/or during pregnancy. Alternatively, some patients may not be apprised of their complete clinical picture and treatment course. A shift towards patient education and collaborative healthcare will continue to improve health literacy and patient participation in their healthcare [37].

The findings in this investigation provide a novel demonstration of disease progression in U.S. alloimmunized pregnancies. The rate of fetal deaths and IUT complications provides a country-specific reflection of disease management and suggests a need for centralizing the conduct of IUTs and defining more structured screening, monitoring and intervention protocols. Obstetric, maternal fetal, neonatal and hematology teams must closely follow these pregnancies through collaborative cross-discipline management to ensure consistent diagnosis, monitoring, and the best possible outcomes for this very treatable disease.

## Figures and Tables

**Table 1:** Participant characteristics.

Demographic and Pregnancy Characteristics	Participants (*N* = 127) *n* (%)
Race and Ethnicity	
Non-Hispanic White	116 (91.3)
Hispanic White	5 (3.9)
Non-Hispanic Black	2 (1.6)
Other	4 (3.1)
Education	
High School or Equivalent	12 (9.4)
Some College	17 (13.4)
Associates Degree or Certificate Program	20 (15.7)
Bachelor’s Degree	40 (31.5)
Master’s Degree	31 (24.4)
Doctoral Degree	7 (5.5)
Insurance	
Private	82 (64.6)
Government Provided	26 (20.5)
Private, Government Provided	3 (2.4)
Military	10 (7.9)
Other	5 (3.9)
None	1 (0.8)
Description of Region of Residence	
Suburban	59 (46.5)
Urban	41 (32.3)
Rural	27 (21.3)
Number of Living Children	
1	11 (8.7)
2	54 (42.9)
3	37 (29.4)
4	12 (9.5)
5+	12 (9.5)
Number of Completed Alloimmunized Pregnancies That Progressed Beyond 12 Weeks Gestation	

1	78 (61.4)
2	33 (26.0)
3	8 (6.3)
4	5 (3.9)
5+	3 (2.4)

**Table 2:** Participant antibody prevalence in titers in pregnancies complicated by red cell alloimmunization.

Characteristic	Alloimmunized Pregnancies with Specific Antibody Known (*n* = 192)	Fetal Antigen Status Known (*n* = 167)	
		Antigen positive (*n* = 131)	Antigen negative (*n* = 36)
	*n* (%)	*n* (%)	*n* (%)
**Single Antibody**	**112 (56.6)**	**71 (54.2)**	**25 (69.4)**
Only Anti-D	27 (24.1)	23 (32.4)	2 (8.0)
Only Anti-E	33 (29.5)	22 (31.0)	7 (28.0)
Only Anti-K	38 (33.9)	18 (25.4)	12 (48.0)
Only Anti-c	6 (5.4)	6 (8.5)	0 (0.0)
**Multiple Antibodies**	**80 (41.7)**	**58 (45.0)**	**13 (34.2)**
Anti-D + Others	41 (51.3)	32 (55.2)	5 (38.5)
Anti-E + Others	34 (42.5)	24 (41.4)	2 (15.4)
Anti-K + Others	15 (18.8)	11 (19.0)	3 (23.1)
Anti-c + Others	23 (28.8)	17 (29.3)	2 (15.4)
	**Titer Status Known (*n* = 176)**	**(*n* = 120)**	**(*n* = 35)**
**Titers Reached Critical Levels**	**125 (71.0)**	**99 (82.5)**	**18 (51.4)**
**Highest Known Titer**			
Anti-K Pregnancies			
<16	18 (34.0)	7 (24.1)	8 (53.3)
16–256	21 (39.6)	12 (41.4)	6 (40.0)
512+	14 (26.4)	10 (34.5)	1 (6.7)
Non-Anti-K Pregnancies			
<16	60 (43.2)	35 (35.0)	13 (56.5)
16–256	63 (45.3)	51 (51.0)	9 (39.1)
512+	16 (11.5)	14 (14.0)	1 (4.3)
**Median Titer**	64.0	64.0	16.0

**Table 3:** Prenatal treatments and pregnancy outcomes in pregnancies complicated by red cell alloimmunization.

		Fetal Antigen Status and Titer Levels Known (*n* = 166)		
Prenatal Treatments	Full Sample (*n* = 200)	Antigen Positive with Critical Titers (*n* = 106)	Antigen Positive with Non- Critical Titers (*n* = 20)	Antigen Negative (*n* = 40)
	*n* (%)	*n* (%)	*n* (%)	*n* (%)
**MCA ^1^ Scan Conducted**				
None	34 (17.1)	6 (5.7)	4 (20.0)	11 (30.6)
Once or Twice	25 (12.5)	7 (6.6)	5 (25.0)	7 (17.5)
Once a Month	18 (9.0)	4 (3.8)	6 (30.0)	3 (7.5)
Once Every Two Weeks	42 (21.1)	26 (24.5)	3 (15.0)	8 (20.0)
Once a Week	80 (40.0)	63 (59.4)	2 (10.0)	10 (25.0)
**IVIG ^2^**	19 (9.5)	15 (14.2)	0 (0.0)	3 (7.5)
**Plasmapheresis**	7 (3.5)	7 (6.6)	0 (0.0	0 (0.0)
**Phenobarbital**	10 (5.0)	9 (8.5)	0 (0.0)	0 (0.0)
**Corticosteroids**	71 (35.5)	53 (50.0)	1 (5.0)	13 (32.5)
**NSTs ^3^**	137 (68.5)	82 (77.4)	13 (65.0)	27 (67.5)
**BPPs ^4^**	99 (49.5)	63 (59.4)	10 (50.0)	16 (40.0)
**IUT ^5^**	45 (22.5)	42 (39.6)	0 (0.0)	1 (2.5)
**Highest MoM ^6^ at First IUT**				
1.01–1.29	1 (2.6)	1 (2.8)	0 (0.0)	0 (0.0)
1.30–1.49	0 (0.0)	0 (0.0)	0 (0.0)	0 (0.0)
1.50–1.69	20 (52.6)	19 (52.8)	0 (0.0)	0 (0.0)
1.70–1.89	10 (26.3)	9 (25.0)	0 (0.0)	1 (100.0)
1.90 or Higher	7 (18.4)	7 (19.4)	0 (0.0)	0 (0.0)
**Number of IUTs**				
1	7 (16.3)	7 (17.1)	0 (0.0)	0 (0.0)
2	12 (27.9)	11 (26.8)	0 (0.0)	1 (100.0)
3	10 (23.3)	10 (24.4)	0 (0.0)	0 (0.0)
4	6 (14.0)	5 (12.2)	0 (0.0)	0 (0.0)
5	5 (11.6)	5 (12.2)	0 (0.0)	0 (0.0)
6+	3 (7.0)	3 (7.3)	0 (0.0)	0 (0.0)
**IUT Complication**	17 (38.6)	17 (40.5)	0 (0.0)	0 (0.0)
**Fetal Complications**				
Anemia on ultrasound	58 (29.0)	52 (49.1)	1 (5.0)	1 (2.5)
Ascites	10 (5.0)	10 (9.4)	0 (0.0)	0 (0.0)
Hydrops	4 (2.1)	4 (4.2)	0 (0.0)	0 (0.0)
Death in Utero	11 (5.5)	10 (9.3)	1 (5.0)	0 (0.0)
Death in Utero Due to HDFN	10 (5.0)	10 (9.4)	0 (0.0)	0 (0.0)

^1^ MCA = Middle Cerebral Artery. ^2^ IVIG = Intravenous Immune Globulin. ^3^ NSTs = Non-Stress Test. ^4^ BPPs = Biophysical Profile. ^5^ IUT = Intrauterine Transfusion. ^6^ MoM = Multiples of the Median.

**Table 4:** Description of pregnancies resulting in death due to HDFN.

Alloimmunized Pregnancy Number; Pregnancy Year	Antibody; Highest Known Titer	Monitoring/Treatments Received	MCA ^1^ Doppler MoM ^2^ at Time of First Transfusion; Gestational Age at First Transfusion	Gestational Age at Death	Patient-Reported Case Description	Subsequent Pregnancy History
First, 2012	Anti-K 1024	MCA Doppler ultrasounds; 1 IUT ^3^	>1.90 18–19 weeks	19 weeks	Patient request for referral, early MCA Dopplers and plasmapheresis and IVIG ^4^ denied. Donor blood added to ascites, baby injected with Lasix, baby showed more heart damage and worse anemia after the IUT, never moved again, died a week later.	Three subsequent antigen positive pregnancies receiving IVIG, plasmapheresis, phenobarbital, MCA Dopplers beginning prior to 16 weeks, IUTs beginning at 24–28 weeks all resulting in live birth.
First, 2014	Anti-D 2048	None	Not conducted Not conducted	24 weeks	Despite patient request, provider did not refer to MFM ^5^ prior to 24 weeks. First MFM appointment 2 days after fetal death in utero.	One subsequent antigen positive pregnancy, MCA Dopplers beginning prior to 16 weeks, IUTs beginning at 16–17 weeks resulting in live birth.
Sixth, 2016	NR 64	MCA Doppler ultrasounds, 1 IUT	1.50–1.69 20–21 weeks	20 weeks	Baby’s MCA Doppler MoM fluctuating for previous 2.5 weeks before ending at 1.69. IUT conducted 48 h later. Upon admission for IUT baby had passed. Autopsy confirmed HDFN ^6^.	One subsequent pregnancy receiving IVIG and plasmapheresis, MCA Dopplers beginning prior to 16 weeks, IUTs beginning at 22–23 weeks resulting in live birth.
Second, 2017	Anti-D 64	MCA Doppler ultrasounds, NSTs ^7^, antenatal corticosteroids, 1 IUT	NR 28–29 weeks	28 weeks	Developed antibodies after missed Rh immune globulin. Patient reports failed IUT due to provider inexperience and hesitancy to refer for procedure.	One subsequent antigen positive pregnancy, IUTs beginning at 30–31 weeks, MCA dopplers beginning prior to 16 weeks, resulting in live birth.
Second, 2018	Anti-D, Anti-C 1024	MCA Doppler ultrasounds; IVIG; 3 IUTs	>1.90 16–17 weeks	20 weeks	IVIG initiated after MCA Doppler MoMs reading over 2.0. Chorioamnionitis following IUT, preterm labor.	No subsequent pregnancies.
Third, 2018	Anti-K, Anti-Fya, Anti-Jka 4096	MCA Doppler ultrasounds, IVIG, NSTs, BPPs ^8^, antenatal corticosteroids, 5 IUTs	>1.90 26–27 weeks	30 weeks	True knot in umbilical cord. Same mother as below.	Two subsequent pregnancies, one reported below resulting in fetal death, final pregnancy with antigen negative fetus.
Fourth, 2019	Anti-K, Anti-Fya, Anti-Jka >16,384	MCA Doppler ultrasounds, IVIG, plasmapheresis, NSTs, BPPs	NR Not conducted	15 weeks	Same mother as above.	See above.
First, 2020	Anti-D 1024	MCA Doppler ultrasounds; 1 IUT	NR 22–23 weeks	23 weeks	Never felt movement again after IUT and baby had passed away at MCA Doppler scan 2 days after IUT.	One subsequent antigen positive pregnancy receiving IVIG, MCA Dopplers beginning prior to 16 weeks, IUTs beginning at 30–31 weeks resulting in live birth.
First, 2020	Anti-E, Anti-K 256	None	Not conducted Not conducted	31 weeks	No monitoring or treatment. Incorrect antigen test ordered for father resulting in inaccurate determination of fetal antigen status.	No subsequent pregnancies.
First, 2021	Anti-K 2048	MCA Doppler ultrasounds; 1 IUT	>1.90 18–19 weeks	19 weeks	NR ^9^	One subsequent antigen positive pregnancy receiving IVIG and plasmapheresis, MCA Dopplers beginning 16–18 weeks, IUTs beginning at 24–25 weeks resulting in live birth.

^1^ MCA = Middle Cerebral Artery. ^2^ MoM = Multiples of the Median. ^3^ IUT = Intrauterine Transfusion. ^4^ IVIG = Intravenous Immune Globulin. ^5^ MFM = Maternal-Fetal Medicine. ^6^ HDFN = Hemolytic Disease of the Fetus and Newborn. ^7^ NSTs = Non-Stress Tests. ^8^ BPPs = Biophysical Profile. ^9^ NR = Not Reported.

**Table 5:** Delivery method, rationale and timing in pregnancies complicated by red cell alloimmunization.

		Fetal Antigen Status Known (*n* = 175)		
	Full Sample (*n* = 200)	Antigen Positive (*n* = 122)	Antigen Positive Receiving IUT ^1^ (*n* = 38)	Antigen Negative (*n* = 40)
	*n* (%)	*n* (%)	*n* (%)	*n* (%)
**Vaginal Delivery**	**125 (66.1)**	**85 (68.5)**	**21 (56.8)**	**21 (52.5)**
	Vaginal induction due to alloimmunization			
Induced Due to Alloimmunization	74 (59.2)	56 (65.9)	19 (90.5)	10 (47.6)
**Cesarean Delivery**	**64 (33.9)**	**39 (31.5)**	**16 (43.2)**	**19 (47.5)**
	Reason for cesarean delivery			
Prior Cesarean	31 (48.4)	15 (38.5)	4 (25.0)	13 (68.4)
Baby Was Not Head Down	12 (18.8)	7 (17.9)	4 (25.0)	4 (21.1)
Non-reassuring Fetal Wellbeing	10 (15.6)	9 (23.1)	4 (25.0)	1 (5.3)
Vaginal Delivery Attempted but Unsuccessful	5 (7.8)	4 (10.3)	0 (0.0)	1 (5.3)
Because of Alloimmunization	14 (21.9)	12 (30.8)	5 (31.3)	2 (10.5)
Elective	3 (4.7)	2 (5.1)	1 (6.3)	1 (5.3)
Other	18 (28.1)	9 (23.1)	6 (37.5)	7 (36.8)
	Emergency cesarean			
**Emergency Cesarean**	**21 (10.5)**	**17 (12.6)**	**10 (23.3)**	**4 (10.0)**
Gestational Age at Birth (Weeks)				
≤33 Weeks	14 (7.5)	11 (9.0)	8 (21.6)	3 (7.5)
34–36 Weeks	46 (24.6)	34 (27.9)	20 (54.1)	9 (22.5)
37–38 Weeks	88 (47.1)	59 (48.4)	9 (24.3)	17 (42.5)
39–40 Weeks	34 (18.2)	16 (13.1)	0 (0.0)	10 (25.0)
41+ Weeks	5 (2.7)	2 (1.6)	0 (0.0)	1 (2.5)

^1^ IUT = intrauterine transfusion.

## Data Availability

Dataset available on request from authors.

## Abbreviations

The following abbreviations are used in this manuscript:

BPP	Biophysical profile
DOAJ	Directory of open access journals
HDFN	Hemolytic disease of the fetus and newborn
IRB	Institutional review board
IUT	Intrauterine blood transfusion
IVIG	Intravenous immunoglobulin
LD	Linear dichroism
MCA	Middle cerebral artery
MDPI	Multidisciplinary Digital Publishing Institute
MoM	Multiples of the median
NR	Not reported
NST	Nonstress test
RBC	Red blood cell
TLA	Three letter acronym
US	United States
USA	United States of America
